# Establishment of an Endogenous *Clostridium difficile* Rat Infection Model and Evaluation of the Effects of *Clostridium butyricum* MIYAIRI 588 Probiotic Strain

**DOI:** 10.3389/fmicb.2018.01264

**Published:** 2018-06-18

**Authors:** Kentaro Oka, Takako Osaki, Tomoko Hanawa, Satoshi Kurata, Emi Sugiyama, Motomichi Takahashi, Mamoru Tanaka, Haruhiko Taguchi, Shigeru Kamiya

**Affiliations:** ^1^Tokyo R&D Center, Miyarisan Pharmaceutical Co., Ltd., Tokyo, Japan; ^2^Department of Infectious Diseases, Kyorin University School of Medicine, Tokyo, Japan; ^3^Research Laboratory, Miyarisan Pharmaceutical Co., Ltd., Nagano, Japan; ^4^Department of Immunology, Kyorin University Faculty of Health Sciences, Tokyo, Japan

**Keywords:** *Clostridium difficile*, *C. difficile* associated diarrhea, *C. difficile* infection model, *Clostridium butyricum*, probiotics

## Abstract

*Clostridium difficile* is well known as an agent responsible for pseudomembranous colitis and antibiotic-associated diarrhea. The hamster model utilizing an oral route for infection of *C. difficile* has been considered to be the standard model for analysis of *C. difficile* infection (CDI) but this model exhibits differences to human CDI, most notably as most hamsters die without exhibiting diarrhea. Therefore, we attempted to develop a new non-lethal and diarrheal rat CDI model caused by endogenous *C. difficile* using metronidazole (MNZ) and egg white. In addition, the effects of probiotic strain *Clostridium butyricum* MIYAIRI 588 (CBM) on CDI were examined using this model. Syrian Golden hamsters received clindamycin phosphate orally at 30 mg/kg on 5 days before challenge with either *C. difficile* VPI10463 (hypertoxigenic strain) or KY34 (low toxigenic clinical isolate). Mortality and the presence of diarrhea were observed twice a day for the duration of the experiment. Wistar rats received 10% egg white dissolved in drinking water for 1 week *ad libitum* following intramuscular administration of 200 mg/kg MNZ twice a day for 3 days. Diarrhea score was determined for each day and fecal water content, biotin concentration, and cytotoxin titer in feces were examined. More than 70% of hamsters orally infected with *C. difficile* died without exhibiting diarrhea regardless of toxigenicity of strain. The rats receiving egg white after MNZ administration developed diarrhea due to overgrowth of endogenous *C. difficile*. This CDI model is non-lethal and diarrheal, and some rats in this model were spontaneously cured. The incidence of diarrhea was significantly decreased in *C. butyricum* treated rats. These results indicate that the CDI model using egg white and MNZ has potentially better similarity to human CDI, and implies that treatment with *C. butyricum* may reduce the risk of CDI.

## Introduction

*Clostridium difficile* is the principal pathogen causing pseudomembranous colitis (PMC) and antibiotic-associated diarrhea (AAD; Bartlett et al., 1978; Kelly et al., 1994; Rupnik et al., 2009). *C. difficile* is a Gram-positive, obligate anaerobic spore forming rod, and its virulence is mainly due to the production of the two large clostridial glucosylating toxins. These are designated toxin A (TcdA) and toxin B (TcdB), which have enterotoxic and cytotoxic activity, respectively. Most pathogenic strains produce both toxins A and B, but some produce only toxin B. Naturally occurring isolates which produce only toxin A have not previously been reported. Some isolates produce a binary toxin (CDT) as an additional toxin, but the role of CDT in *C. difficile* infection (CDI) is not well understood (Drudy et al., 2007; Lyras et al., 2009; Rupnik et al., 2009; Kuehne et al., 2010). Toxins A and B target the cytosolic Rho GTPases and modify these enzymes by mono-*O*-glucosylation, leading to host cell morphological changes, secretion inhibition and cell death (Kelly and LaMont, 2008; Chen et al., 2015).

*Clostridium difficile* is an opportunistic pathogen which is occasionally isolated from the intestine or stool of healthy humans as well as being widely distributed in the environment (e.g., river and sea water and soil). Many animals including pigs, cattle, poultry, dogs, horses, rabbits, cats, and goats have been reported as reservoirs for *C. difficile* (al Saif and Brazier, 1996; Janezic et al., 2014; Usui et al., 2014). In humans, the detection rates of *C. difficile* from stool vary depending on age, and are reported to be 1.9–15.4% for adults (Nakamura et al., 1981; Aronsson et al., 1985) and 28.9–61.2% for infants (Viscidi et al., 1981; Kato et al., 1994). Although infants exhibit high prevalence rates for *C. difficile*, most are asymptomatic and a similar phenomenon has been reported in pigs (Usui et al., 2014). An experiment using newborn rabbit ileum demonstrated a lack of toxin A receptors (Eglow et al., 1992), and this has been used as a controversial reason for asymptomatic *C. difficile* carriage in infants. However, these findings have not been replicated in pigs and hamsters and have yet to be investigated in humans. In newborn hamsters, which also do not develop CDI as neonates, significant binding of toxin throughout the intestine has been reported and other factors such as *C. difficile* colonization resistance have been suggested (Keel and Songer, 2007). Therefore, it is presumed that the sensitivity to *C. difficile* toxins and/or the resistance to colonization by *C. difficile* depends on age and the animal species.

The main etiology of CDI in almost all animals is dysbiosis of intestinal microbiota, mainly caused by antibiotic treatment, which causes overgrowth and toxin production by *C. difficile*. As such, antibiotics are used to disrupt microbiota to produce CDI models (except for germ-free animal models). A number of animal species, such as hamsters, guinea pigs, rabbits, mice, rats, and germ-free mice and rats, have been used as CDI models (Chen et al., 2008). The most widely used model utilizes hamsters pretreated with various antibiotics followed by oral challenge with *C. difficile*. In this model, a toxigenic *C. difficile* grows and produces toxins mainly in the cecum, and the hamsters quickly die from severe enterocolitis. This hamster model has been considered to be the standard for analysis of CDI. However, this model exhibits significant differences to human CDI since the symptoms are fulminant and lethal and most hamsters die without exhibiting diarrhea. The course of disease in gnotobiotic mice and rats is also similar to the hamster model (Kamiya et al., 1997). Therefore, the development of non-lethal and diarrheal CDI models is needed in order to fully understand the pathogenic mechanisms of *C. difficile.*

*Clostridium butyricum* is a Gram-positive, obligately anaerobic spore forming rod similar to *C. difficile*. It is also widely distributed in soil and has been isolated from fecal samples from healthy humans (Finegold et al., 1983). *C. butyricum* has been used as a prescription/over the counter probiotic in humans and as a feed additive for animals in Japan and other Asian countries for several decades (Jonsson and Conway, 1992; Seki et al., 2003). *C. butyricum* MIYAIRI588 (CBM) strain is a well-known clostridial probiotic strain also approved as a feed additive by the European Food Safety Authority. It has previously been reported that CBM has various effects on health and disease, including anti-inflammatory and anti-infectious activity including an antagonistic activity on *C. difficile* (Takahashi et al., 2000, 2004; Nakanishi et al., 2003; Woo et al., 2011; Hayashi et al., 2013). CBM has been shown to protect against lethal CDI in germ-free mice but its effect on mild CDI symptoms is still not clear.

During the course of evaluating hamster and rat CDI models, we found that a commercially available SPF rat strain harbors an endogenous *C. difficile* which is toxigenic but did not induce a similar cecal distribution CDI. We therefore tried to establish a rat CDI model caused by this endogenous *C. difficile* with the hope that it would be non-lethal and diarrheal and therefore more similar to human CDI. Additionally, the effect of CBM on this model was examined.

## Materials and Methods

### Bacterial Strain

*Clostridium difficile* VPI 10463 (hypertoxigenic strain) or KY 34 (low toxigenicity clinical isolate) was used for infection models. *C. butyricum* MIYAIRI 588 was used as a probiotic strain.

### Experimental Infection

Experimental infections were performed under the control of the ethical committee at Kyorin University or Miyarisan Pharmaceutical Co., Ltd. by experimental location, and Japanese Government Animal Protection and Management Law. Approval numbers at Kyorin University and Miyarisan were No. 06-31-055 for the hamster experiment and No. 2008-1 for the rat experiment, respectively.

### Hamster CDI Model by *C. difficile* Challenge

Syrian Golden hamsters (SPF, 5 weeks old, 80–90 g, male) were purchased from Japan SLC, Inc. (Hamamatsu, Japan). Two hamsters were housed in a polypropylene cage, and one group of hamsters in this experiment were kept in a positive air pressured vinyl isolator to prevent cross-contamination. All food, drinking water, bedding, cages, and isolators were sterilized before use. The bedding was changed to new sterilized one every 2 days. Hamsters received clindamycin phosphate (Dalacin S Injection, Pfizer Japan, Inc., Tokyo, Japan) orally at 30 mg/kg of body weight on 5 days before oral challenge with 6.0 × 10^7^ cfu of *C. difficile* VPI10463 spores or 3.1 × 10^7^ cfu of *C. difficile* KY 34 spores. Mortality and the presence of diarrhea were observed twice a day for duration of the experiment.

### Rat CDI Model by *C. difficile* Challenge

Wistar rats (SPF, 9 weeks old, 180–200 g, male) were purchased from Japan SLC, Inc. Housing procedure was similar to the hamster experiment described above, except using appropriate cage size and sterilized paper towels under sterilized stainless mesh floors instead of bedding to prevent coprophagy. Rats received 200 mg/kg of metronidazole (MNZ) twice a day for 3 days intramuscularly before challenge with *C. difficile* VPI10463 spores at a dose of 2.9 × 10^7^ cfu/rat. The presence of diarrhea was observed every day and the severity of the diarrhea was scored using a scale described previously (Kurita et al., 2000): 0 (normal; normal stool or absent); 1 (slight; slightly wet and soft stool); 2 (moderate, wet and unformed stool with moderate perianal staining of the coat); and 3 (severe, watery stool with severe perianal staining of the coat). At the same time, stool was sampled and the number of *C. difficile* was determined by culture method as described in Section “Materials and Methods.”

### Rat CDI Model Using Endogenous *C. difficile* and the Effects of *C. butyricum*

Wistar rats (SPF, 9 weeks old, 180–200 g, male) were obtained from Japan SLC, Inc., and housed in the same manner as described above. The rats received 10% egg white spray dried (Wako Pure Chemicals Industries, Ltd., Osaka, Japan) dissolved in their drinking water for 1 week *ad libitum* following intramuscular administration of 200 mg/kg MNZ twice a day for 3 days. Sterilized normal saline was inoculated orally twice a day for 1 week during the administration of egg white for controls. Instead of normal saline, 5.1 × 10^7^ cfu of CBM strain spores were inoculated in the same manner to examine the effectiveness of the probiotic in this model (CBM group). The presence of diarrhea was observed every day and the severity of the diarrhea was scored using the scale described above. Stool was collected at same time and the number of *C. difficile, C. butyricum*, and *Bacteroides* was examined. Fecal water content, fecal cytotoxin titer, and fecal biotin concentration were also measured.

### Quantification of *C. difficile, C. butyricum*, and *Bacteroides* in Feces

The numbers of viable *C. difficile, C. butyricum*, and *Bacteroides* in 1 g of feces were quantified by culture method. The feces were suspended and diluted in phosphate buffer containing 0.05% L-cystain hydrochloride hydrate (Sigma–Aldrich, St. Louis, MO, United States) and 0.05% Tween80 (Wako Pure Chemicals Industries, Ltd.), and 50 μL of appropriate diluent was spread onto agar medium and cultured. CCFA agar (OXOID, Hampshire, United Kingdom) supplemented with 0.1% sodium taurocholate (Wako Pure Chemicals Industries, Ltd.), *C. butyricum*-selective medium (Sato and Tanaka, 1997), and NBGT agar (Mitsuoka, 1978) were used for selective counting for *C. difficile, C. butyricum*, and *Bacteroides*, respectively. An anaerobic chamber (10% CO_2_, 10% H_2_, and N_2_ to balance) was used for anaerobic manipulation and cultivation.

### Isolation, Identification, and Typing of *C. difficile*

In the experiment using the rat CDI model, we performed tests to identify and type *C. difficile* isolates, since endogenous *C. difficile* was detected from the feces in the non-infected control group. *C. difficile* isolates were identified by PCR assay according to the procedure previously described with slight modifications (Gumerlock et al., 1991; Kato et al., 1998). Single colonies obtained during *C. difficile* enumeration were suspended in 100 μL of TE [10 mM Tris-HCl, 1 mM EDTA (pH 8.0)] and boiled for 10 min. After centrifugation at 10,000 ×*g* for 5 min, the resultant supernatant was used as template DNA. PCR ribotyping of the *C. difficile* isolate was carried out using the same template DNA according to methods described previously (Oka et al., 2012), modified from previous studies (Kato et al., 1991, 2001).

### Cytotoxicity Assay

Cytotoxicity was determined using African green monkey kidney (Vero) cells by the method described previously (Kamiya and Borriello, 1992; Oka et al., 2012). For a comparison of cytotoxicity of *C. difficile* strains, the culture supernatant of *C. difficile* in brain–heart infusion (BHI) broth incubated at 37°C for 48 h was used as the sample. For the examination of toxin production in biotin-limited conditions, defined medium (Yamakawa et al., 1996) supplemented with an arbitrary concentration of biotin was used. Overnight culture of *C. difficile* in defined medium containing 50 nM biotin was 1000-fold diluted with sterile saline and inoculated into new defined medium containing the arbitrary concentration of biotin. After incubation at 37°C for 5 days, the culture supernatant was used as the sample. For fecal cytotoxicity assay, feces were diluted with Dulbecco’s phosphate buffered saline followed by centrifugation and filtration, and the resultant supernatant was used as the sample. For the cytotoxin production experiment using *C. difficile* in the presence of *C. butyricum* culture supernatant, *C. difficile* SLC, which was the endogenous strain isolated from Wistar rats in this study, was cultured in BHI broth supplemented with 0, 50, and 100% concentration of *C. butyricum* culture supernatant to investigate the effect of *C. butyricum* on cytotoxin production. The cytotoxicity of the sample was determined as the highest dilution resulting in a 100% cell rounding after incubation for 24 h.

### Quantification of Biotin in Feces

Biotin content in feces was measured by a bioassay using *Lactobacillus plantarum* ATCC 8014. Biotin assay medium “Nissui” (Nissui pharmaceutical, Tokyo, Japan) was used according to the manufacturer’s instructions. Free form biotin in feces was extracted with water at 60°C for 10 min and the fecal suspension was centrifuged and filtered using a 0.45 μm pore-size filter. Some biotin exists in samples in the protein-bound form, and therefore to measure total biotin the feces was treated with 4.5N H_2_SO_4_ at 121°C for 1 h using an autoclave then neutralized with the same volume of 4.5 N NaOH to release bound biotin then assayed as above.

### Fecal Water Content

Day 3 rat fecal samples were weighed and centrifuged at 8,000 ×*g* for 10min, and then the supernatant was discarded. The weight loss by centrifugation was defined as fecal free water content. The feces was then freeze-dried and weighed. The weight loss by freeze-drying was defined as fecal bound water content.

### Statistics

For the statistical comparison of the diarrhea score, fecal water content, bacterial counts in feces, fecal cytotoxicity, and fecal biotin concentration, Student’s *t*-test or Mann–Whitney’s *U*-test was used for parametric and non-parametric data, respectively. For the incidence of diarrhea, Fisher’s exact probability test was used. Quantitative data were expressed as mean with SD or SE of the mean, and the normality of a distribution and homogeneity of variances were evaluated by chi square goodness-of-fit test and Bartlett’s test.

## Results

### Hamster CDI Model by *C. difficile* Challenge

**FIGURE 1:**
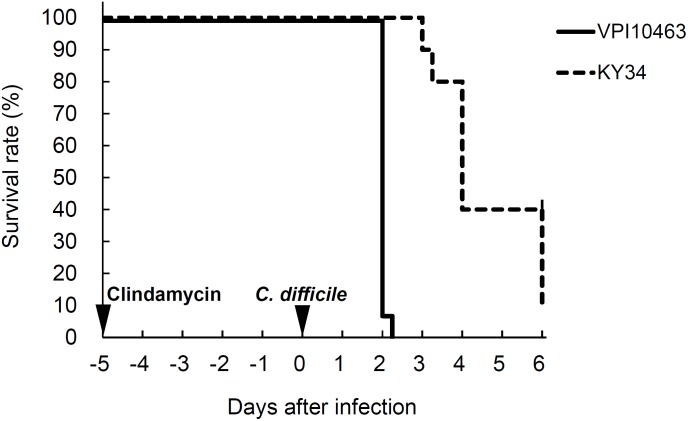
Survival rates of hamsters infected with *C. difficile* VPI10463 or KY34 strains.

All of the hamsters infected with *C. difficile* VPI10463 strain died within 2 days. On the other hand, the hamsters infected with KY34 strain died on day 3 at the earliest. The survival curve was delayed compared with VPI10463 and 90% of hamsters died within 6 days (**Figure 1**). Cytotoxic titer in the culture supernatant of KY34 strain (toxin titer, <2^6^) was more than 256 times lower than that of VPI10463 strain (toxin titer, 2^14^). Incidence of diarrhea was only 10–20%, but almost all hamsters orally infected with *C. difficile* died without exhibiting diarrhea regardless of toxigenicity of strain (**Table 1**).

**Table 1:** Mortality and the incidence of diarrhea of hamsters after oral administration of *C. difficile*.

Strain	Number of dead hamsters/number of hamsters tested (mortality)	Number of hamsters with diarrhea/number of hamsters tested (incidence of diarrhea)
*C. difficile* VPI10463	30/30 (100%)	3/30 (10%)
*C. difficile* KY34	9/10 (90%)	2/10 (20%)

### Rat CDI Model by *C. difficile* Challenge

**FIGURE 2:**
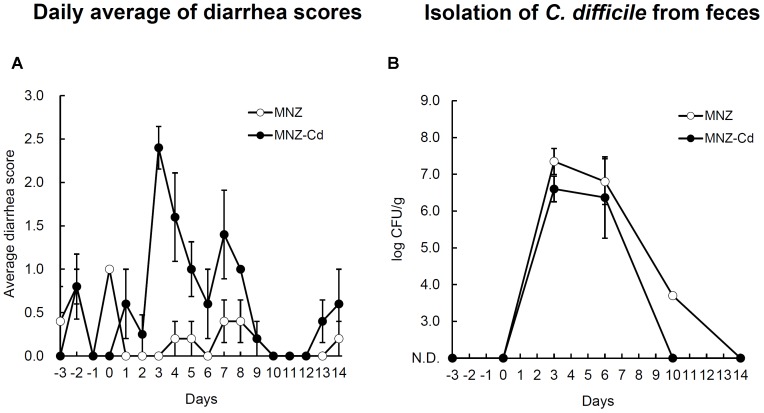
Daily average diarrhea scores **(A)** and the number of *C. difficile* in feces **(B)** of rats orally infected with *C. difficile* VPI10463. MNZ indicates the control group treated with only metronidazole. MNZ-Cd indicates the *C. difficile-*infected and MNZ treated group. Data are expressed as mean ± SD of the mean. Error bars indicate SDs.

**FIGURE 3:**
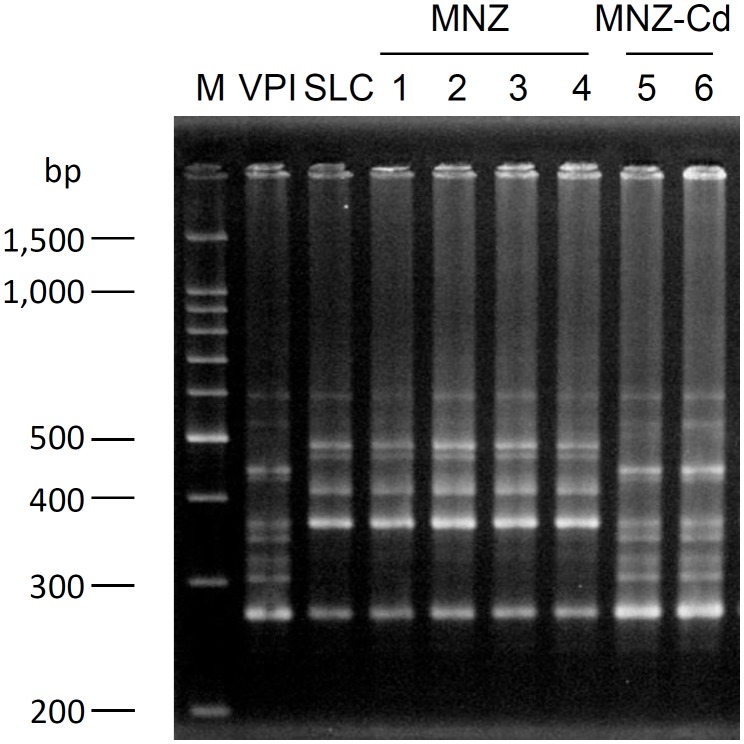
PCR ribotype pattern of *C. difficile* isolated from rat feces. Lane M, 100 bp ladder marker; Lane VPI, *C. difficile* VPI 10463; Lane SLC, *C. difficile* isolated from feces of non-infected rat, Lane 1–4, *C. difficile* isolated from feces of Group MNZ rat; and Lane 5 and 6, *C. difficile* isolated from feces of Group MNZ-Cd rat.

Rats infected with VPI10463 strain after treatment with MNZ exhibited diarrhea from day 3 and the symptoms were spontaneously cured with time (**Figure 2A**). On the other hand, rats not infected with *C. difficile* exhibited no diarrhea (**Figure 2A**), although *C. difficile* was isolated from both groups and there was no difference in the bacterial cell numbers in feces between the two groups (**Figure 2B**). The results of PCR ribotyping demonstrated that the isolate from the uninfected rat group was not the VPI10463 strain, instead deemed to be an endogenous strain named SLC (**Figure 3**). *C. difficile* SLC strains were repeatedly isolated from rats purchased from the same supplier on different days (data not shown). The cytotoxic titer in culture supernatants of SLC strain (toxin titer, 2^8^) was 128 times lower than that of VPI10463 strain (toxin titer, 2^15^).

### Rat CDI Model Using Endogenous *C. difficile* and the Effects of *C. butyricum*

**FIGURE 4:**
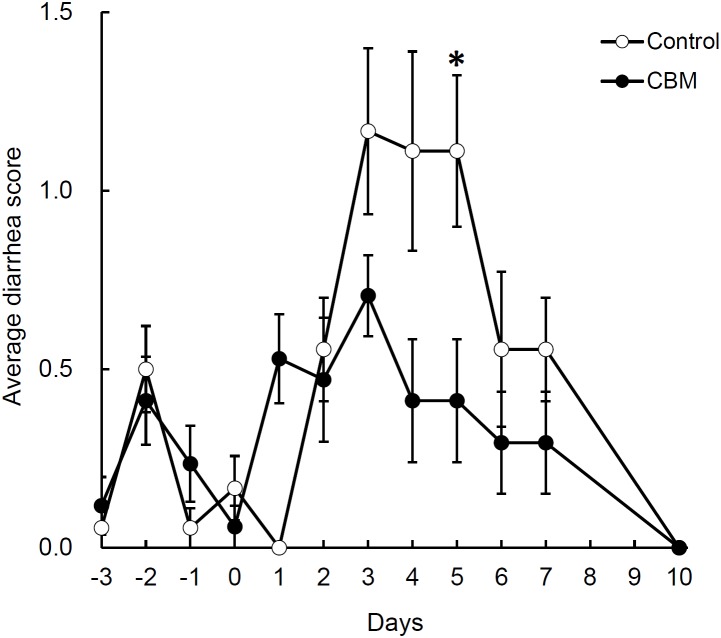
Effect of CBM on diarrhea. Data are expressed as means. Error bars indicate SE of the mean. Asterisk indicates statistical significance by Mann–Whitney’s *U* test with *p* = 0.019.

**FIGURE 5:**
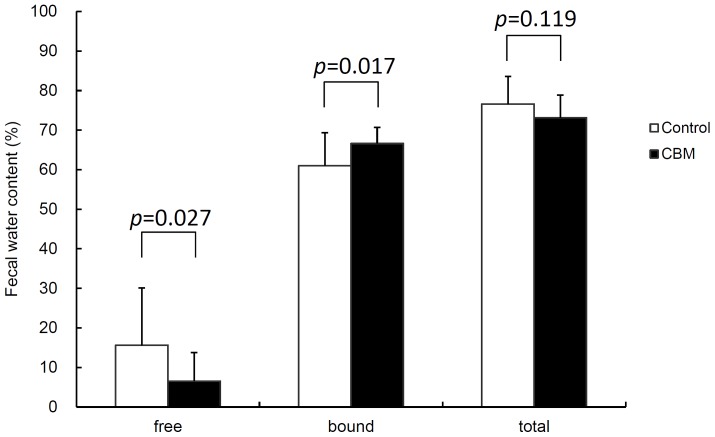
Effect of CBM on fecal water content at day 3. Data are expressed as means. Error bars indicate SDs. Student’s *t*-test was used for statistical comparison.

**FIGURE 6:**
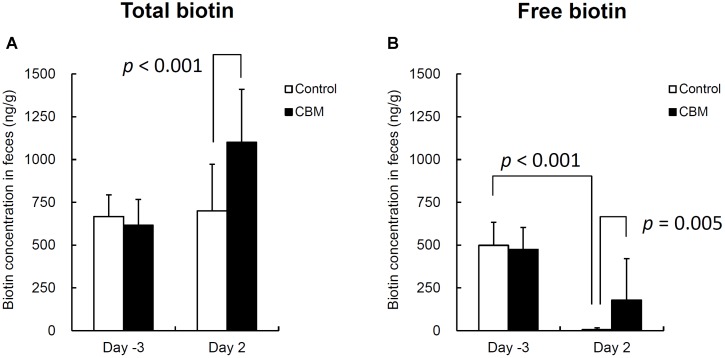
Total **(A)** and free **(B)** biotin concentrations in rat feces. Data are expressed as means. Error bars indicate SDs. Student’s *t*-test was used for statistical comparison.

**FIGURE 7:**
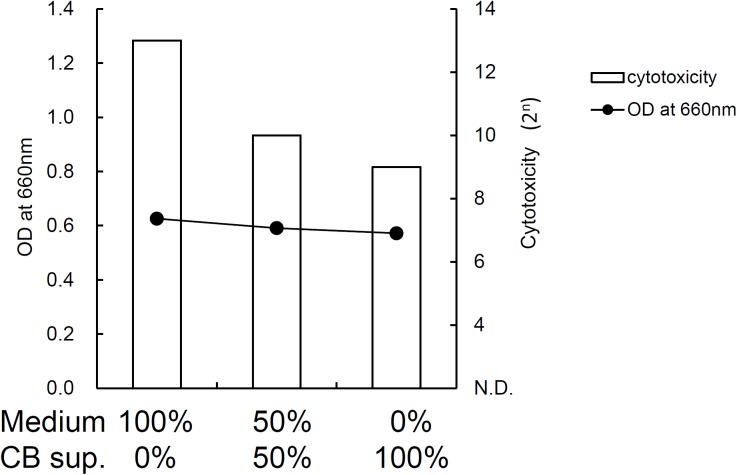
Effect of *C. butyricum* culture supernatant on cytotoxin production (cytotoxicity) and growth (OD at 660 nm) of *C. difficile* SLC *in vitro.* Medium indicates BHI broth with pH adjusted to 7.0. CB sup. indicates culture supernatant of *C. butyricum* cultured in BHI broth for 24 h followed by pH adjustment to 7.0.

Toxin production of SLC strain was enhanced in biotin-limited conditions (toxin titers were <2^2^, 2^8^, and 2^6^ at 50 nM, 50 pM, and 50 fM of biotin, respectively) as similarly demonstrated previously (Yamakawa et al., 1996). Given these results, we presumed that we could make a rat CDI model using these endogenous *C. difficile* with a spontaneous cure by administering egg white containing avidin which binds biotin. We also subsequently evaluated the efficacy of the probiotic CBM in this model. Rats treated with MNZ and egg white developed diarrhea from day 3 and then spontaneously cured (**Figure 4**). In the control group, the incidence of watery diarrhea (score 3) throughout the observation period was 16.7% and the incidence of watery and/or moderate diarrhea (score ≥ 2) was 66.7% (**Table 2**). This was significantly reduced by administration of *C. butyricum* (**Figure 4** and **Table 2**). In addition, fecal-free water significantly reduced (**Figure 5**). *C. difficile* was isolated from feces of both groups and there were no difference in the bacterial counts. On the other hand, depletion of *Bacteroides* in feces caused by MNZ was significantly improved on day 2 by *C. butyricum* treatment (**Table 3**). Cytotoxin titer in feces of rats treated with *C. butyricum* on day 3 (mean ± SEM in log_2_ scale, 10 ± 0.3) was lower than that of the control group (mean ± SEM in log_2_ scale, 11 ± 0.4). The concentration of free form biotin in feces was significantly decreased by administration of egg white (**Figure 6**) and oral treatment with *C. butyricum* increased both free and total biotin concentration in feces on day 2 (**Figure 6**). Culture supernatants of *C. butyricum* inhibited the toxin production of *C. difficile* SLC strain but did not affect the bacterial counts (optical density at 660 nm; **Figure 7**).

**Table 2:** Effect of CBM on incidence of diarrhea.

	Score 3	≥Score 2
Control	3/18 (16.7%)^∗^	12/18 (66.7%)
CBM	0/17 (0%)	5/17 (29.4%)
*p*-value	0.125	0.030

*^∗^Number of rats exhibiting diarrhea/number of rats tested (%). Fisher’s exact probability test was used for statistical comparison.*

**Table 3:** Number (detection rate) of *C. difficile, C. butyricum*, and *Bacteroides* in feces of rats.

		Log cfu/g of feces (detection rate)		
		Day -3	Day 0	Day 2
*C. difficile*	Control	N.D.	N.D.	6.3 ± 2.0 (12/18)^∗^
	CBM	N.D.	6.1 (1/17)	6.2 ± 1.4 (10/17)
*C. butyricum*	Control	N.D.	N.D.	N.D.
	CBM	N.D.	N.D.	6.5 ± 1.6 (15/17)
*Bacteroides*	Control	8.1 ± 0.2 (18/18)	4.3 ± 1.1 (18/18)	5.5 ± 1.7 (18/18)
	CBM	8.0 ± 0.2 (17/17)	4.8 ± 1.5 (17/17)	8.1 ± 1.3^∗∗^ (17/17)

*Data are expressed as mean ± SD. ^∗^Number of rats positive for the indicated bacteria/number of rats tested. ^∗∗^Statistically significant with *p* < 0.001 by Student’s-test. N.D., not detected (<2.3 log cfu/g or <200 cfu/g, less than 1 colony per 50 μl of 10-fold fecal suspension).*

## Discussion and Conclusion

In this study, we tried to establish a rat non-lethal and diarrheal CDI model using endogenous *C. difficile* which is spontaneously cured without any treatment. Furthermore, we attempted to evaluate the efficacy of a probiotic strain, CBM, by using this model. First, we evaluated the standard hamster model which is prepared by pretreatment with antibiotics followed by *C. difficile* challenge. In previous reports, the antibiotic most often used was clindamycin by intragastric, subcutaneous, or intraperitoneal administration, and the interval between the antibiotic treatment and *C. difficile* challenge was 1–5 days (Swanson et al., 1991; Kink and Williams, 1998; Sambol et al., 2001; Anton et al., 2004). The challenge dose of *C. difficile* was 10^1^–10^4^ per hamster in one report (Sambol et al., 2001) and 10^4^–10^6^ in the remaining three (Swanson et al., 1991; Kink and Williams, 1998; Anton et al., 2004). Therefore, we compared mortalities up to 5 days after administration of intragastric clindamycin and a dose range of 10^4^–10^7^ *C. difficile* VPI10463 spores as a preliminary test. When 2.3 × 10^4^, 3.7 × 10^6^, and 4.4 × 10^7^ spores of *C. difficile* were administered at 1 day after clindamycin treatment, mortality was 0% (0/31), 38.7% (12/31), and 30.0% (9/30), and incidence of diarrhea was 0% (0/31), 3.2% (1/31), and 3.3% (1/30), respectively, at 6 days after infection. On the other hand, when the interval was prolonged to 5 days, all hamsters administered with 10^7^ spores died within 2 days of infection (**Figure 1** and **Table 1**). This result indicates that sufficient dysbiosis of cecal microbiota by clindamycin to allow *C. difficile* overgrowth requires a few days. For the next preliminary test, we compared the mortality to a less toxigenic *C. difficile* strain KY 34 [screened from 73 clinical isolates by cytotoxic activity against Vero cells (data not shown)]. Although the cytotoxic activity of KY 34 was more than 256 times lower than that of VPI10463, there was no difference in the mortality except the survival curves. Furthermore, since the incidence of diarrhea in both cases was very low, it is presumed that the hamster model is very sensitive to *C. difficile* toxin making it a poor parallel to human CDI. However, a limitation of our data is that we have no comparative data at lower doses of *C. difficile*. Sambol et al. (2001) reported that a toxin A-negative, toxin B-positive strain induced asymptomatic colonization and produced diarrhea followed by subsequent spontaneous recovery in a hamster at 10^2^ cfu *C. difficile*, although three epidemic and one asymptomatic toxigenic strains showed similar lethal effects in the same conditions. Therefore, the observation of a small difference in disease between the two strains in our study may be due to the higher numbers of *C. difficile* administered or less likely due to study design factors.

We next tried to establish a non-lethal and diarrheal CDI model by using rats, as we theorized that there may have been differences in *C. difficile* sensitivity depending on the animal species. As a result of comparative examination using several kinds of antibiotics, including intramuscular MNZ, oral ceftriaxone, and intraperitoneal cyclophosphamide, we selected intramuscular MNZ pretreatment, which most reliably induced diarrhea after oral infection by *C. difficile* VPI10463 strain (data not shown). In this model, all the rats infected with *C. difficile* exhibited diarrhea by day 3 (5/5 heads) and the symptoms spontaneously cured with time (**Figure 2A**). When the interval between MNZ pretreatment and CDI was prolonged to 3 days from 1 day, only 60% (3/5 heads) of rats suffered diarrhea. This result indicates that the dysbiosis by MNZ might be easier to recover than that caused by clindamycin treatment in hamsters. In the control group which received only MNZ, no rats had diarrhea during a same experimental period. However, interestingly, almost the same numbers of *C. difficile* were recovered from feces of both groups (**Figure 2B**). As a result of PCR ribotyping, we confirmed that the strain derived from control rats was an endogenous strain and named it SLC (**Figure 3**). The cytotoxicity of this strain was 128 times lower than that of VPI10463 and it was suggested that the SLC strain did not cause diarrhea due to its low toxigenicity.

Yamakawa et al. (1996) reported that toxin production of *C. difficile* was enhanced in biotin-limited conditions. Based on these observations, we speculated that endogenous *C. difficile* could induce diarrhea if the concentration of biotin in the rat intestine could be decreased, and therefore we tried administration of egg white which contains avidin. As we expected, toxin production of *C. difficile* SLC was enhanced in biotin-limited conditions similar to previous *in vitro* reports and depletion of free biotin in the feces of avidin treated rats was observed (**Figure 6B**). The rats exhibited diarrhea from 3 days after the end of MNZ treatment and spontaneously recovered with time in a similar way to the *C. difficile* VPI10463 infection model. This model has an improved similarity to human CDI compared with other animal models given its non-lethality and diarrhea symptoms, although it needs the atypical condition of biotin depletion.

Next, we used this model to evaluate the efficacy of the probiotic CBM on CDI. CBM was found to be able to successfully suppress average diarrhea scores, the incidence of diarrhea, and the fecal-free and bound water content, although the decrease in diarrhea scores was relatively small. To assess the degree of dysbiosis and restoration of intestinal microbiota, we measured the number of *Bacteroides* in feces as a representative indicator, since it is known that MNZ has high activity against anaerobes (Löfmark et al., 2010) and *Bacteroides* is the dominant anaerobe in rats (Nakanishi et al., 2003). After the intramuscular administration of MNZ for 3 days, the number of *Bacteroides* in feces was dramatically decreased 1000-fold. Following this, the numbers of *Bacteroides* spontaneously restored by day 2 up to 10^5^ cfu/g of feces but still remained 100-fold lower than normal levels. In the CBM treated group, restoration of the number of *Bacteroides* up to normal level was observed, indicating that CBM may promote the restoration of the intestinal microbiota (**Table 3**) although entire microbiome analysis (e.g., meta-16S analysis) is needed to confirm this. On the other hand, since there was no difference in the number of *C. difficile* at day 2 (**Table 3**), it was suggested that the effects of CBM were not due to growth inhibition of *C. difficile* but suppression of toxin production or toxin activity. This view is supported by a previous report demonstrating an inhibitory effect on *C. difficile* toxin production by CBM (Woo et al., 2011). Consistent with this, cytotoxicity in the feces of the CBM treated group was lower than that of the control group at day 3, although there was no statistical significance due to experimental problems. At the beginning of this study, we were not expecting to need to quantify toxin in feces, and therefore we had no alternative but to use the remaining fecal samples after measurement of fecal water content (which only included the solid residue after centrifugation discarding the supernatant). In *in vitro* culture experiments, CBM culture supernatant did not affect the growth of *C. difficile* but did decrease the cytotoxicity in a dose-dependent manner (**Figure 7**). Along with this, CBM also statistically significantly increased both total and free biotin concentrations in feces at day 2 (**Figure 6**), which may be another mechanism attenuating *C. difficile* toxin production. We presume that the increase in biotin concentration either derived from CBM itself or promotion of other biotin-producing commensal bacteria. Draft genome sequencing of CBM has revealed that it has a biotin synthetic pathway with adenosylmethionine-8-amino-7-oxononanoate transaminase (EC 2.6.1.62), dethiobiotin synthase (EC 6.3.3.3), and biotin synthase (EC 2.8.1.6) (data not shown).

In this study, we have successfully established a rat CDI model using an endogenous *C. difficile* strain and have used it to evaluate the efficacy of a probiotic strain (although evaluation using average diarrheal scores is not necessarily a sufficient test for probiotics). This model is induced by antibiotic treatment and is spontaneously cured without any treatment, which has more parallels to human CDI. This may make it a useful animal system in addition to the existing hamster and mouse CDI models. Artificial infection using laboratory or clinical strains and use or not of avidin allows the model to be modified depending on purpose. It is expected that our findings can provide a new tool for better understanding of the pathoetiology of CDI and improve prevention and treatment.

## Author Contributions

KO, TO, TH, SaK, MoT, HT, and ShK contributed to data analysis and interpretation, methodology development, conception, and design for hamster experiment. KO, ES, MoT, MaT, HT, and ShK contributed to data analysis and interpretation, methodology development, conception, and design for rat experiment. KO contributed to manuscript writing. KO and ShK reviewed the paper, prepared figures, wrote, and improved the scientific quality of the manuscript. All authors read and approved the final manuscript.

## Conflict of Interest Statement

The authors declare that the research was conducted in the absence of any commercial or financial relationships that could be construed as a potential conflict of interest, with the exception of Miyarisan Pharmaceutical.

## References

[B1] al Saif N. Brazier J. S. (1996). The distribution of *Clostridium difficile* in the environment of South Wales. *J. Med. Microbiol.* 45 133–137. 10.1099/00222615-45-2-133 8683549

[B2] Anton P. M. O’Brien M. Kokkotou E. Eisenstein B. Michaelis A. Rothstein D. (2004). Rifalazil treats and prevents relapse of *Clostridium difficile*-associated diarrhea in hamsters. *Antimicrob. Agents Chemother.* 48 3975–3979. 10.1128/AAC.48.10.3975-3979.2004 15388461 PMC521872

[B3] Aronsson B. Möllby R. Nord C. E. (1985). Antimicrobial agents and *Clostridium difficile* in acute enteric disease: epidemiological data from Sweden, 1980-1982. *J. Infect. Dis.* 151 476–481. 10.1093/infdis/151.3.476 3973405

[B4] Bartlett J. G. Chang T. W. Gurwith M. Gorbach S. L. Onderdonk A. B. (1978). Antibiotic-associated pseudomembranous colitis due to toxin-producing clostridia. *N. Engl. J. Med.* 298 531–534. 10.1056/NEJM197803092981003 625309

[B5] Chen S. Sun C. Wang H. Wang J. (2015). The role of Rho GTPases in toxicity of *Clostridium difficile* toxins. *Toxins* 7 5254–5267. 10.3390/toxins7124874 26633511 PMC4690124

[B6] Chen X. Katchar K. Goldsmith J. D. Nanthakumar N. Cheknis A. Gerding D. N. (2008). A mouse model of *Clostridium difficile*-associated disease. *Gastroenterology* 135 1984–1992. 10.1053/j.gastro.2008.09.002 18848941

[B7] Drudy D. Fanning S. Kyne L. (2007). Toxin A-negative, toxin B-positive *Clostridium difficile*. *Int. J. Infect. Dis.* 11 5–10. 10.1016/j.ijid.2006.04.003 16857405

[B8] Eglow R. Pothoulakis C. Itzkowitz S. Israel E. J. O’Keane C. J. Gong D. (1992). Diminished *Clostridium difficile* toxin A sensitivity in newborn rabbit ileum is associated with decreased toxin A receptor. *J. Clin. Invest.* 90 822–829. 10.1172/JCI115957 1325998 PMC329936

[B9] Finegold S. M. Sutter V. L. Mathisen G. E. (1983). “Normal indigenous intestinal flora,” in *Human Intestinal Microflora in Health and Disease*, ed. Hentges D. J. (New York, NY: Academic Press), 3–31. 10.1016/B978-0-12-341280-5.50007-0

[B10] Gumerlock P. H. Tang Y. J. Meyers F. J. Silva J. Jr. (1991). Use of the polymerase chain reaction for the specific and direct detection of *Clostridium difficile* in human feces. *Rev. Infect. Dis.* 13 1053–1060. 10.1093/clinids/13.6.1053 1775837

[B11] Hayashi A. Sato T. Kamada N. Mikami Y. Matsuoka K. Hisamatsu T. (2013). A single strain of *Clostridium butyricum* induces intestinal IL-10-producing macrophages to suppress acute experimental colitis in mice. *Cell Host Microbe* 13 711–722. 10.1016/j.chom.2013.05.013 23768495

[B12] Janezic S. Zidaric V. Pardon B. Indra A. Kokotovic B. Blanco J. L. (2014). International *Clostridium difficile* animal strain collection and large diversity of animal associated strains. *BMC Microbiol.* 14:173. 10.1186/1471-2180-14-173 24972659 PMC4100527

[B13] Jonsson E. Conway P. (1992). “Probiotics for pigs,” in *Probiotics, the Scientific Basis*, ed. Fuller R. (London: Chapman and Hall), 259–316.

[B14] Kamiya S. Borriello S. P. (1992). A non-haemagglutinating form of *Clostridium difficile* toxin A. *J. Med. Microbiol.* 36 190–197. 10.1099/00222615-36-3-190 1548692

[B15] Kamiya S. Taguchi H. Yamaguchi H. Osaki T. Takahashi M. Nakamura S. (1997). Bacterioprophylaxis using *Clostridium butyricum* for lethal caecitis by *Clostridium difficile* in gnotobiotic mice. *Rev. Med. Microbiol.* 8(Suppl.1), S57–S59. 10.1097/00013542-199712001-00029

[B16] Kato H. Kato N. Watanabe K. Iwai N. Nakamura H. Yamamoto T. (1998). Identification of toxin A-negative, toxin B-positive *Clostridium difficile* by PCR. *J. Clin. Microbiol.* 36 2178–2182. 9665986 10.1128/jcm.36.8.2178-2182.1998 PMC105000

[B17] Kato H. Kato N. Watanabe K. Ueno K. Ushijima H. Hashira S. (1994). Application of typing by pulsed-field gel electrophoresis to the study of *Clostridium difficile* in a neonatal intensive care unit. *J. Clin. Microbiol.* 32 2067–2070. 7814526 10.1128/jcm.32.9.2067-2070.1994 PMC263943

[B18] Kato H. Kita H. Karasawa T. Maegawa T. Koino Y. Takakuwa H. (2001). Colonisation and transmission of *Clostridium difficile* in healthy individuals examined by PCR ribotyping and pulsed-field gel electrophoresis. *J. Med. Microbiol.* 50 720–727. 10.1099/0022-1317-50-8-720 11478676

[B19] Kato N. Ou C. Y. Kato H. Bartley S. L. Brown V. K. Dowell V. R. Jr. (1991). Identification of toxigenic *Clostridium difficile* by the polymerase chain reaction. *J. Clin. Microbiol.* 29 33–37. 1993763 10.1128/jcm.29.1.33-37.1991 PMC269697

[B20] Keel M. K. Songer J. G. (2007). The distribution and density of *Clostridium difficile* toxin receptors on the intestinal mucosa of neonatal pigs. *Vet. Pathol.* 44 814–822. 10.1354/vp.44-6-814 18039894

[B21] Kelly C. P. LaMont J. T. (2008). *Clostridium difficile* - more difficult than ever. *N. Engl. J. Med.* 359 1932–1940. 10.1056/NEJMra0707500 18971494

[B22] Kelly C. P. Pothoulakis C. LaMont J. T. (1994). *Clostridium difficile* colitis. *N. Engl. J. Med.* 330 257–262. 10.1056/NEJM199401273300406 8043060

[B23] Kink J. A. Williams J. A. (1998). Antibodies to recombinant *Clostridium difficile* toxins A and B are an effective treatment and prevent relapse of *C. difficile*-associated disease in a hamster model of infection. *Infect. Immun.* 66 2018–2025. 9573084 10.1128/iai.66.5.2018-2025.1998 PMC108158

[B24] Kuehne S. A. Cartman S. T. Heap J. T. Kelly M. L. Cockayne A. Minton N. P. (2010). The role of toxin A and toxin B in *Clostridium difficile* infection. *Nature* 467 711–713. 10.1038/nature09397 20844489

[B25] Kurita A. Kado S. Kaneda N. Onoue M. Hashimoto S. Yokokura T. (2000). Modified irinotecan hydrochloride (CPT-11) administration schedule improves induction of delayed-onset diarrhea in rats. *Cancer Chemother. Pharmacol.* 46 211–220. 10.1007/s002800000151 11021738

[B26] Löfmark S. Edlund C. Nord C. E. (2010). Metronidazole is still the drug of choice for treatment of anaerobic infections. *Clin. Infect. Dis.* 50(Suppl.1), S16–S23. 10.1086/647939 20067388

[B27] Lyras D. O’Connor J. R. Howarth P. M. Sambol S. P. Carter G. P. Phumoonna T. (2009). Toxin B is essential for virulence of *Clostridium difficile*. *Nature* 458 1176–1179. 10.1038/nature07822 19252482 PMC2679968

[B28] Mitsuoka T. (1978). *Intestinal Bacteria and Health.* Tokyo: Harcourt Brace Jovanovich Japan.

[B29] Nakamura S. Mikawa M. Nakashio S. Takabatake M. Okado I. Yamakawa K. (1981). Isolation of *Clostridium difficile* from the feces and the antibody in sera of young and elderly adults. *Microbiol. Immunol.* 25 345–351. 10.1111/j.1348-0421.1981.tb00036.x 7253967

[B30] Nakanishi S. Kataoka K. Kuwahara T. Ohnishi Y. (2003). Effects of high amylose maize starch and *Clostridium butyricum* on metabolism in colonic microbiota and formation of azoxymethane-induced aberrant crypt foci in the rat colon. *Microbiol. Immunol.* 47 951–958. 10.1111/j.1348-0421.2003.tb03469.x 14695445

[B31] Oka K. Osaki T. Hanawa T. Kurata S. Okazaki M. Manzoku T. (2012). Molecular and microbiological characterization of *Clostridium difficile* isolates from single, relapse, and reinfection cases. *J. Clin. Microbiol.* 50 915–921. 10.1128/JCM.0558811 22205786 PMC3295180

[B32] Rupnik M. Wilcox M. H. Gerding D. N. (2009). *Clostridium difficile* infection: new developments in epidemiology and pathogenesis. *Nat. Rev. Microbiol.* 7 526–536. 10.1038/nrmicro2164 19528959

[B33] Sambol S. P. Tang J. K. Merrigan M. M. Johnson S. Gerding D. N. (2001). Infection of hamsters with epidemiologically important strains of *Clostridium difficile*. *J. Infect. Dis.* 183 1760–1766. 10.1086/320736 11372028

[B34] Sato R. Tanaka M. (1997). Intestinal distribution and intraluminal localization of orally administered *Clostridium butyricum* in rats. *Microbiol. Immunol.* 41 665–671. 10.1111/j.1348-0421.1997.tb01909.x 9343816

[B35] Seki H. Shiohara M. Matsumura T. Miyagawa N. Tanaka M. Komiyama A. (2003). Prevention of antibiotic-associated diarrhea in children by *Clostridium butyricum* MIYAIRI. *Pediatr. Int.* 45 86–90. 10.1046/j.1442-200X.2003.01671.x 12654076

[B36] Swanson R. N. Hardy D. J. Shipkowitz N. L. Hanson C. W. Ramer N. C. Fernandes P. B. (1991). In vitro and in vivo evaluation of tiacumicins B and C against *Clostridium difficile*. *Antimicrob. Agents Chemother.* 35 1108–1111. 10.1128/AAC.35.6.1108 1929250 PMC284295

[B37] Takahashi M. Taguchi H. Yamaguchi H. Osaki T. Kamiya S. (2000). Studies of the effect of *Clostridium butyricum* on *Helicobacter* pylori in several test models including gnotobiotic mice. *J. Med. Microbiol.* 49 635–642. 10.1099/0022-1317-49-7-635 10882089

[B38] Takahashi M. Taguchi H. Yamaguchi H. Osaki T. Komatsu A. Kamiya S. (2004). The effect of probiotic treatment with *Clostridium butyricum* on enterohemorrhagic *Escherichia coli* O157:H7 infection in mice. *FEMS Immunol. Med. Microbiol.* 41 219–226. 10.1016/j.femsim.2004.03.010 15196571

[B39] Usui M. Nanbu Y. Oka K. Takahashi M. Inamatsu T. Asai T. (2014). Genetic relatedness between Japanese and European isolates of *Clostridium difficile* originating from piglets and their risk associated with human health. *Front. Microbiol.* 5:513. 10.3389/fmicb.2014.00513 25339943 PMC4189341

[B40] Viscidi R. Willey S. Bartlett J. G. (1981). Isolation rates and toxigenic potential of *Clostridium difficile* isolates from various patient populations. *Gastroenterology* 81 5–9. 7239125

[B41] Woo T. D. Oka K. Takahashi M. Hojo F. Osaki T. Hanawa T. (2011). Inhibition of the cytotoxic effect of *Clostridium difficile* in vitro by Clostridium butyricum MIYAIRI 588 strain. *J. Med. Microbiol.* 60(Pt 11), 1617–1625. 10.1099/jmm.0.033423-0 21700738

[B42] Yamakawa K. Karasawa T. Ikoma S. Nakamura S. (1996). Enhancement of *Clostridium difficile* toxin production in biotin-limited conditions. *J. Med. Microbiol.* 44 111–114. 10.1099/00222615-44-2-111 8642571

